# Impact of Obesity on Port Insertion Time in Gynecologic Laparoscopy Using the Open Technique: A Retrospective Study

**DOI:** 10.7759/cureus.101760

**Published:** 2026-01-18

**Authors:** Yutoku Shi, Mia Maeda, Sachiyo Sugino, Kiyoshi Niiya, Kotaro Ichida

**Affiliations:** 1 Obstetrics and Gynecology, Kobe City Medical Center West Hospital, Kobe, JPN

**Keywords:** laparoscopic surgery, obesity, thickness of subcutaneous fat tissue, trocar insertion, wound closure time

## Abstract

Introduction

Laparoscopic surgery is increasingly performed for benign gynecologic ovarian tumors in Japan. Laparoscopic surgery for obese patients is sometimes challenging due to their increased abdominal wall thickness. The aim of this study was to evaluate whether obesity affects the time of primary trocar insertion and related perioperative outcomes in gynecologic laparoscopic surgery using the open (Hasson) technique.

Methods

This retrospective cohort study included 85 patients who underwent laparoscopic surgery for benign adnexal tumors at Kobe City Medical Center West Hospital, Kobe, Japan, between January 2023 and August 2025. Patients were classified into the normal-weight group (BMI 18.5-24.9 kg/m², n=65) and the obese group (BMI ≥25 kg/m², n=20). The primary outcome was the time of primary trocar insertion. Secondary outcomes included wound closure time, thickness of subcutaneous fat tissue and umbilical wall, complication rates, and correlations among these variables. Correlations among operative times and anatomical parameters were explored using Spearman’s rank correlation. A mixed-effects linear model was applied to evaluate the association between BMI and primary trocar insertion time while adjusting for the surgeon as a random effect and case order as a fixed effect.

Results

The median primary trocar insertion time was four minutes (range, 1-21) in the normal-weight group and six minutes (range 2-20) in the obese group. Patients with obesity had significantly thicker subcutaneous fat tissue (median 35 mm (range 15-51) vs 20 mm (7-44), p<0.01) and umbilical wall thickness (median 21 mm (6-45) vs 10 mm (3-31), p<0.01). In a mixed-effects linear model accounting for surgeon and case order, higher BMI showed a non-significant trend toward longer primary trocar insertion time (0.17 minutes per BMI unit; 95% CI, −0.01 to 0.34; p = 0.06), while case order did not significantly affect primary port insertion time (−0.075 minutes per case; 95% CI, −0.17 to 0.02; p = 0.11). Spearman’s analysis showed a moderate correlation between subcutaneous fat tissue and umbilical wall thickness (ρ=0.63, p<0.01). Complication rates did not differ significantly (normal-weight 10.8% vs. obese 20%, p=0.28).

Conclusion

In this retrospective cohort, laparoscopic access using the open technique was performed in normal-weight and obese patients without significant prolongation of primary trocar insertion. Obese patients had greater subcutaneous fat and umbilical wall thickness, but these did not clearly increase technical difficulty after accounting for surgeon-related factors. While not conclusive evidence of procedural equivalence, the findings suggest that minimally invasive access can be achievable in selected obese patients with a standardized technique and careful preoperative assessment. Surgeon experience remains an important determinant of access outcomes. Prospective studies are needed to confirm these observations and refine evidence-based guidance for laparoscopic entry in patients with elevated BMI.

## Introduction

In recent years, laparoscopic surgery has become more common in Japan. Most gynecologic ovarian benign tumors are resected by laparoscopic surgery [1]. Even in obese patients, laparoscopic surgery is less invasive than open surgery and is associated with a lower risk of surgical site infection (SSI) and shorter hospital stays [2]. However, laparoscopic surgery is sometimes challenging for patients with obesity. In clinical practice, the author sometimes experiences that increased abdominal wall thickness leads to a prolonged time required for laparoscopic port insertion.

Although several studies have investigated obesity in relation to port insertion methods [3], few have specifically analyzed port insertion time. A previous small study reported a port insertion time of about five minutes using the open technique [4]. However, another study reported that trocar insertion in obese patients may require specific technical considerations and can be associated with increased difficulty in laparoscopic manipulation; nevertheless, laparoscopic surgery can be performed safely with appropriate preoperative assessment and preparation [5].

Therefore, in this study, we sought to evaluate whether differences exist in port insertion time and abdominal wall thickness between patients with and without obesity when using the open (Hasson) technique.

## Materials and methods

Study design and participants

This was a retrospective cohort study conducted at the Kobe City Medical Center West Hospital, Kobe, Japan, comparing the surgical outcome of patients with normal body mass index (BMI) and patients with obesity, who underwent gynecologic laparoscopy surgery. Medical records and surgical records were reviewed to extract perioperative variables and postoperative outcomes.

Patients were assigned to two groups according to the preoperative BMI: a normal-weight group (BMI 18.5 to <25.0 kg/m^2^) and an obese group (BMI ≥25 kg/m^2^). The cut-off point of the BMI of the two groups was in accordance with the Asian-specific recommendation by the Japan Society for the Study of Obesity (JASSO) [6].

Patients were eligible if they were over 16 years of age and underwent gynecologic laparoscopic surgery of the adnexa. Inclusion criteria were a preoperative diagnosis of a benign adnexal tumor and the availability of preoperative CT or MRI imaging. Exclusion criteria included postoperative pathological diagnosis of malignancy, intraoperative confirmation of a non-adnexal tumor, pregnancy, and missing key outcome data.

Characteristics, time of primary trocar insertion, wound closure time, thickness of subcutaneous fat tissue, thickness of umbilical wall, and the rate of surgical complications were compared between the two groups.

Sample size

Since there were no previous reports describing the time of primary trocar insertion, including mean and standard deviation (SD), for patients with normal weight and obesity by open technique procedures, sample size calculation was challenging. The plan was to collect approximately 100 samples, assuming that 20% of the study participants would be excluded based on the exclusion criteria. From January 2023 to August 2025, 124 patients met the study criteria.

Perioperative management

When the patient was scheduled for surgery, preoperative imaging tests such as CT or MRI were performed. A pelvic examination and transvaginal sonography checkup were done before discharge. Subsequent outpatient follow-up was scheduled within one month.

Surgical procedure

General anesthesia was applied to all patients with laparoscopic surgery. The primary abdominal trocar was created at the umbilicus. The open (Hasson) technique was used to access the peritoneal cavity. A skin incision of approximately 1.5-2.0 cm was made at the umbilicus, and the subcutaneous tissue and fascia were dissected using Metzenbaum scissors, and the peritoneum was opened under direct visualization. After confirmation of entry into the peritoneal cavity, pneumoperitoneum was started. Port replacement was at diamond configuration position, a 12 mm trocar (Kii Balloon Blunt Tip System; Applied Medical Resources Corporation, Rancho Santa Margarita, California, United States) at umbilical position, and three additional port sites were 5 mm diameter at the lower abdomen (Kii Advanced Fixation Sleeve CFF05). Pneumoperitoneum was maintained at 10 mmHg during the surgery. In this study, the surgical procedures were performed by a board-certified obstetrician-gynecologist (without endoscopic surgical certification and with less than 15 years of clinical experience) as the primary surgeon, with a first assistant with more than 20 years of experience and certified in gynecologic endoscopic surgery. Although the first assistant had provided advice when necessary, the primary progression and execution of the surgical procedures were performed by the primary surgeon. Measurements were performed by the anesthesiologist and operating room nurse and documented in the anesthesia record.

Definitions

Time of primary trocar insertion was defined as the time from umbilical skin incision to successful establishment of pneumoperitoneum, confirmed by laparoscopic visualization of intra-abdominal organs at a stable pneumoperitoneum pressure of 10 mmHg. Wound closure time was defined as the time from removal of the final trocar with cessation of pneumoperitoneum to completion of suturing of all port-site wounds. The thickness of the subcutaneous fat tissue was measured around the umbilicus and defined as the distance from the skin surface to the rectus fascia on CT or MRI images. Umbilical wall thickness was defined as the vertical distance from the umbilical skin to the peritoneum, measured on CT or MRI images.

Outcome (endpoints and assessment)

The primary endpoint was the time required for primary trocar insertion, obtained from the anesthetic record. Secondary endpoints included wound closure time, postoperative complications within one month, and the correlations among primary trocar insertion time, wound closure time, BMI, subcutaneous fat thickness, and umbilical wall thickness.

Learning curve

To evaluate surgeon-specific learning curves, case order was defined as the chronological sequence of surgeries performed by each surgeon. Learning curves were visualized by plotting the time of primary trocar insertion against case order for each surgeon separately. In addition, the effect of surgical experience on operative performance was assessed using linear mixed-effects models, in which case order was included as a fixed effect and surgeon identity as a random intercept to account for inter-surgeon variability.

Statistical analysis

Because the data did not follow a normal distribution, continuous variables are primarily presented as medians with ranges, and non-parametric tests were applied for group comparisons. The Mann-Whitney U test was used to compare continuous variables between the normal-weight and obese groups, and categorical variables were compared using Fisher’s exact test. At the same time, mean ± SD was also reported as supplementary descriptive statistics. This facilitated comparison with previous studies and provided useful information for future research planning, including sample size estimation, particularly in studies focusing on laparoscopic port insertion techniques. 

To account for potential confounding by surgeon-related factors, including inter-surgeon variability and learning-curve effects, a mixed-effects model was performed. The time of primary trocar insertion was used as the dependent variable. BMI and case order within each surgeon were included as fixed effects, and surgeon was included as a random intercept. Ninety-five percent confidence intervals (95% CIs) were calculated. Correlation analyses between anatomical variables (BMI, thickness of subcutaneous fat tissue, thickness of umbilical wall ) and time of primary trocar insertion, wound closure time were performed using Spearman’s rank correlation as exploratory analyses.

All tests were two-sided, and a p-value <0.05 was considered statistically significant. Data were analyzed by using EZR (version 1.68; Saitama Medical Center, Jichi Medical University, Saitama, Japan), a graphical user interface for R [7].

Ethical approval

The study protocol was approved by the Institutional Review Board of Kobe City Medical Center West Hospital (approval number: 25-020). The required written informed consent was obtained before proceeding with the surgery.

## Results

Analysis of characteristics

**Figure 1:**
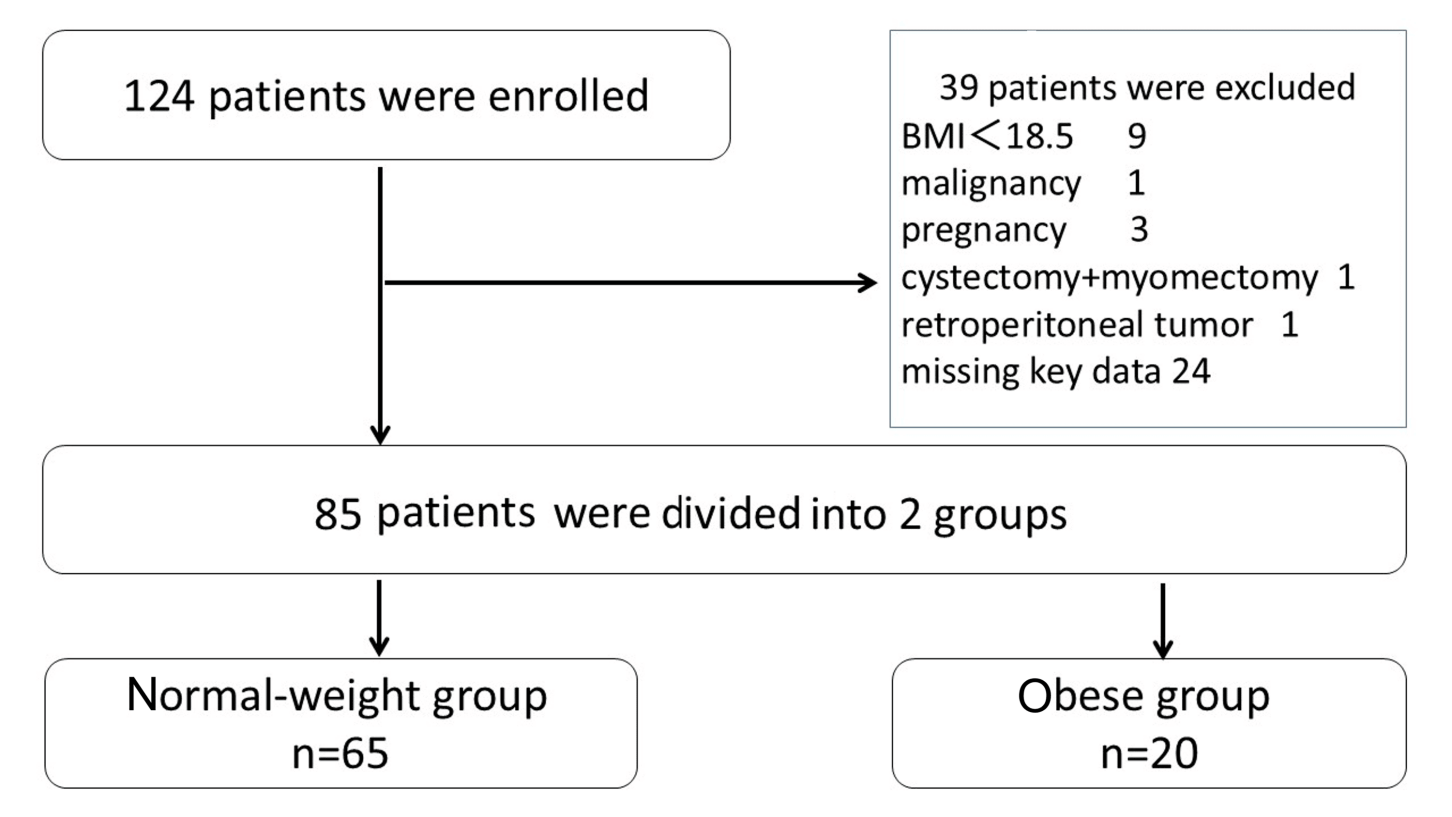
Flowchart of study patient selection

A total of 124 patients were included in this study. Of these, 39 were excluded; nine with BMI less than 18.5, one with malignancy in pathology, three with surgery during pregnancy, one with an additional procedure of laparoscopic myomectomy, one with a retroperitoneal tumor confirmed during surgery, and 24 cases with incomplete key data. The remaining 85 patients were divided into two groups: the normal-weight group and the obese group. The patient selection flowchart is shown in Figure 1.

**Figure 2:**
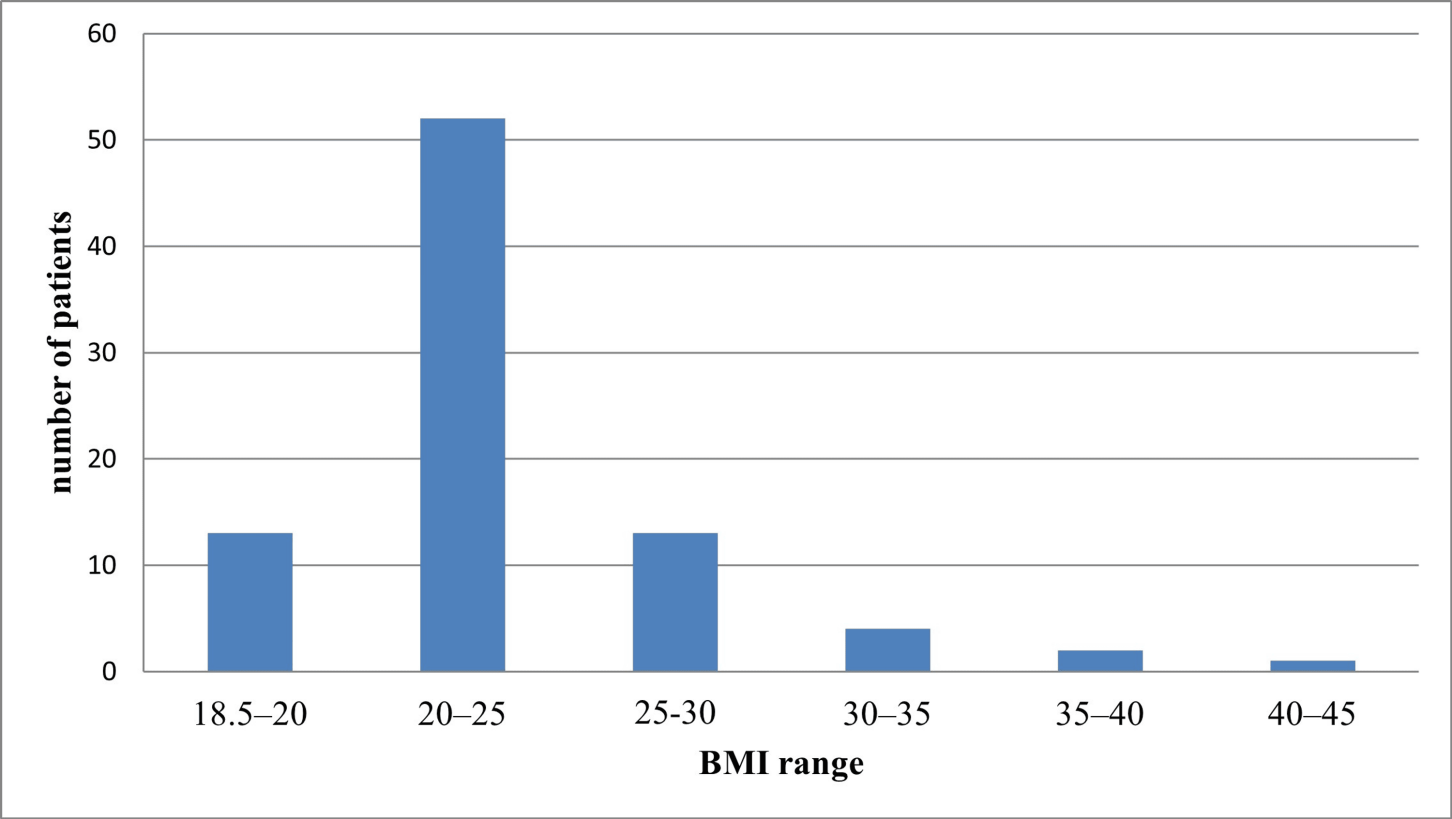
BMI distribution of study patients BMI: body mass index

A histogram of the BMI distribution of study participants is given in Figure 2. The number of patients with BMI 18.5-20 kg/m^2^ was 13 (15.3%), BMI 20-25 kg/m^2^ was 52 (61.2%), BMI 25-30 kg/m^2^ was 13 (15.3%), BMI 30-35 kg/m^2^ was four (4.7%), BMI 35-40 kg/m^2^ was two (2.4%), and BMI 40-45 kg/m^2^ was one (1.2%).

Characteristics of the groups are listed in Table 1. Age, BMI, gravida, para, history of previous cesarian section (CS), number of previous CS, history of previous abdominal surgery except CS, number of previous abdominal surgery except CS, history of diabetes mellitus, type of laparoscopic surgery performed, and pathological diagnoses were compared. There was no statistical significance for these characteristics between the two groups.

**Table 1:** Characteristics of patients Continuous variables were analyzed by Mann-Whitney U test, categorical variables were analyzed by Fisher's exact test. CS: cesarian section

Characteristics	Normal-weight group (n=65)	Obese group (n=20)	p-value
Age (years), median (range)	39 (17-81)	43 (24-83)	0.27
BMI (kg/m^2^), median (range)	20.9 (18.6-24.8)	29.2 (25.3-43.0)	<0.01
Gravida, median (range)	1 (0-7)	1 (0-3)	0.41
Para, median (range)	1 (0-3)	0 (0-3)	0.60
History of previous CS, n (%)	5 (7.7%)	1 (5.0%)	>0.99
Number of previous CS, median (range)	0 (0-2)	0 (0-1)	0.67
History of previous abdominal surgery except CS, n (%)	9 (13.8%)	4 (20%)	0.49
Number of previous abdominal surgeries, except CS, median (range)	0 (0-1)	0 (0-1)	0.51
History of diabetes mellitus, n (%)	3 (4.6%)	1 (5.0%)	>0.99
Type of operation, n (%)			
Cystectomy	38 (58%)	8 (40%)	0.20
Salpingo-oophorectomy	27 (42%)	13 (65%)	0.078
Salpingectomy	1 (1.5%)	0 (0%)	>0.99
Pathological diagnosis, n (%)			
Serous cystadenoma	18 (28%)	7 (35%)	0.58
Mucinous cystadenoma	2 (3.1%)	0 (0%)	>0.99
Fibroma	0 (0%)	2 (10%)	0.053
Endometriotic cyst	15 (23%)	4 (20%)	>0.99
Mature cystic teratoma	26 (40%)	7 (35%)	0.80
Ectopic pregnancy	1 (1.5%)	0 (0%)	>0.99
Borderline tumor	3 (4.6%)	0 (0%)	>0.99

The median age (range) of patients was 39 (17-81) years in the normal-weight group and 43 (24-83) years in the obese group. Five out of 65 patients in the normal weight group had a history of CS, compared to one out of 20 in the obese group. Nine patients in the normal weight group had a history of abdominal surgery except CS, compared to four in the obese group. There was no statistical difference for rate of diabetes mellitus. Type of operations were laparoscopic cystectomy, salpingo-oophorectomy, and salpingectomy. Unilateral cystectomy and the opposite side of salpingo-oophorectomy were performed in two cases, one in the normal-weight group, one in the obese group.

Postoperative pathological diagnoses included serous cystadenoma, mucinous cystadenoma, fibroma, endometriotic cyst, mature cystic teratoma, ectopic pregnancy, and ovarian borderline tumor.

Surgeon characteristics and learning curves

A total of five surgeons (Surgeon A-E) participated in this study, with Surgeon A performing nine cases, Surgeon B 18 cases, Surgeon C 24 cases, Surgeon D 10 cases, and Surgeon E 24 cases. All of the surgeons had less than 15 years of clinical experience. Surgeon A had performed more than 50 gynecologic laparoscopic procedures prior to this study, whereas the other four surgeons each had experience with more than 100 such procedures. Table 2 summarizes surgeon characteristics, the cumulative order of cases performed during the study period, and the corresponding primary trocar insertion times for each surgeon.

**Table 2:** Surgeon characteristics, cumulative case order during the study period, and time of primary trocar insertion

Surgeon	Years of clinical experience	Experienced case number (n)	Time of primary trocar insertion (minutes), median (range)
A	6	9	5 (2-21)
B	14	18	6 (1-17)
C	10	24	4 (2-12)
D	11	10	3 (2-20)
E	7	24	4 (2-15)

**Figure 3:**
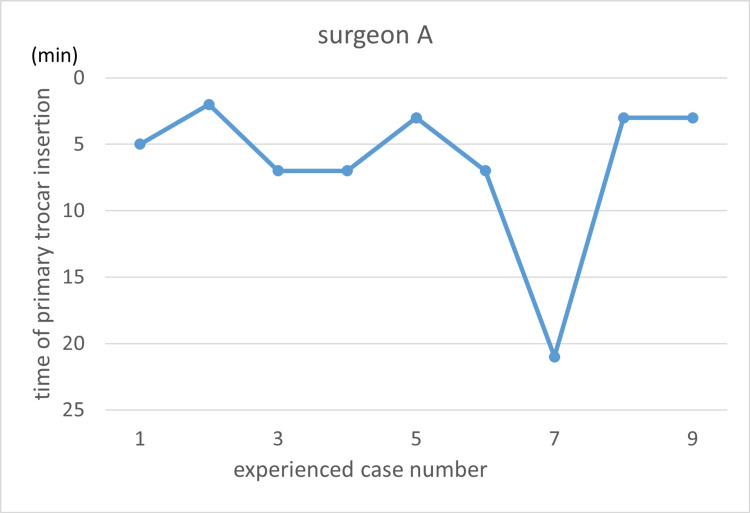
Learning curve of Surgeon A

**Figure 4:**
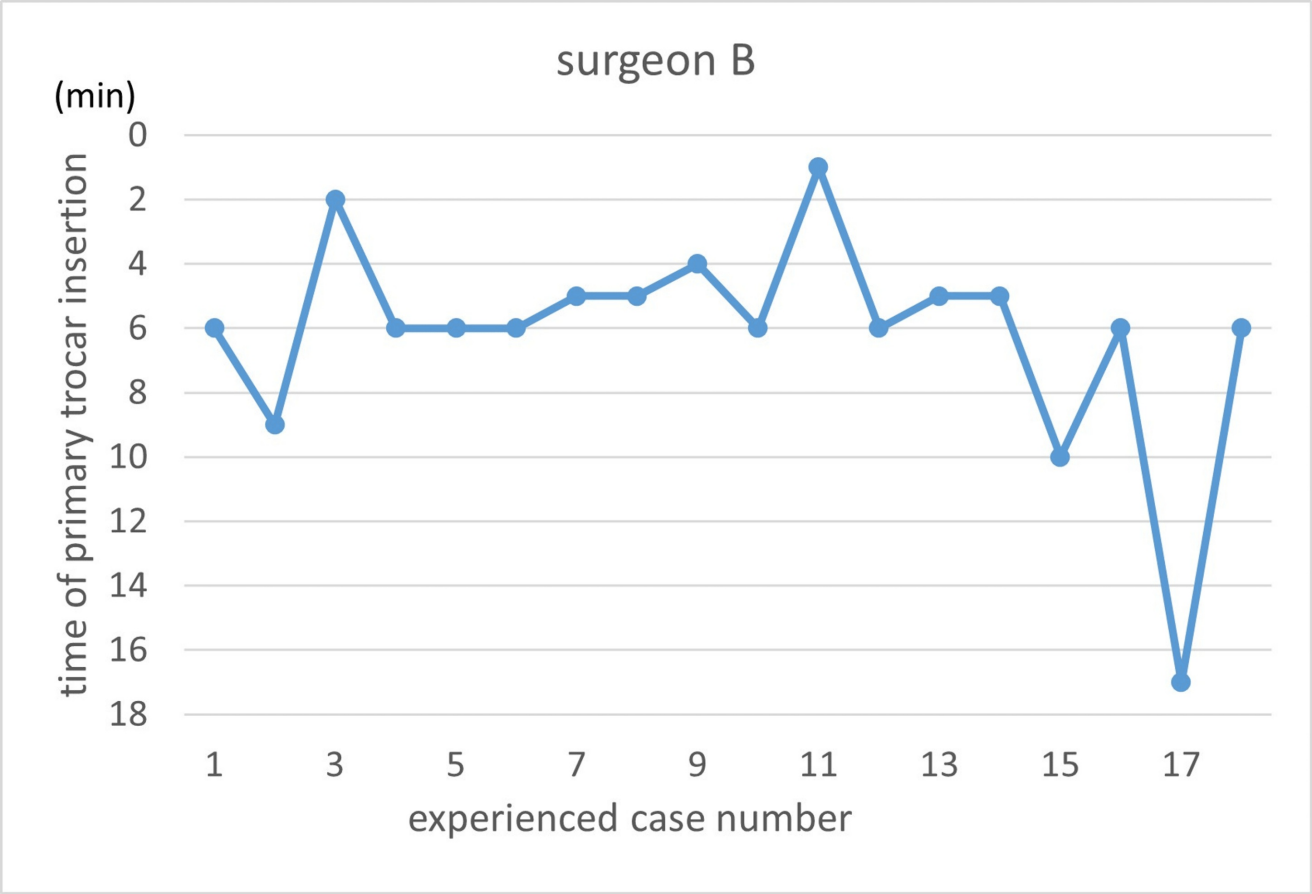
Learning curve of Surgeon B

**Figure 5:**
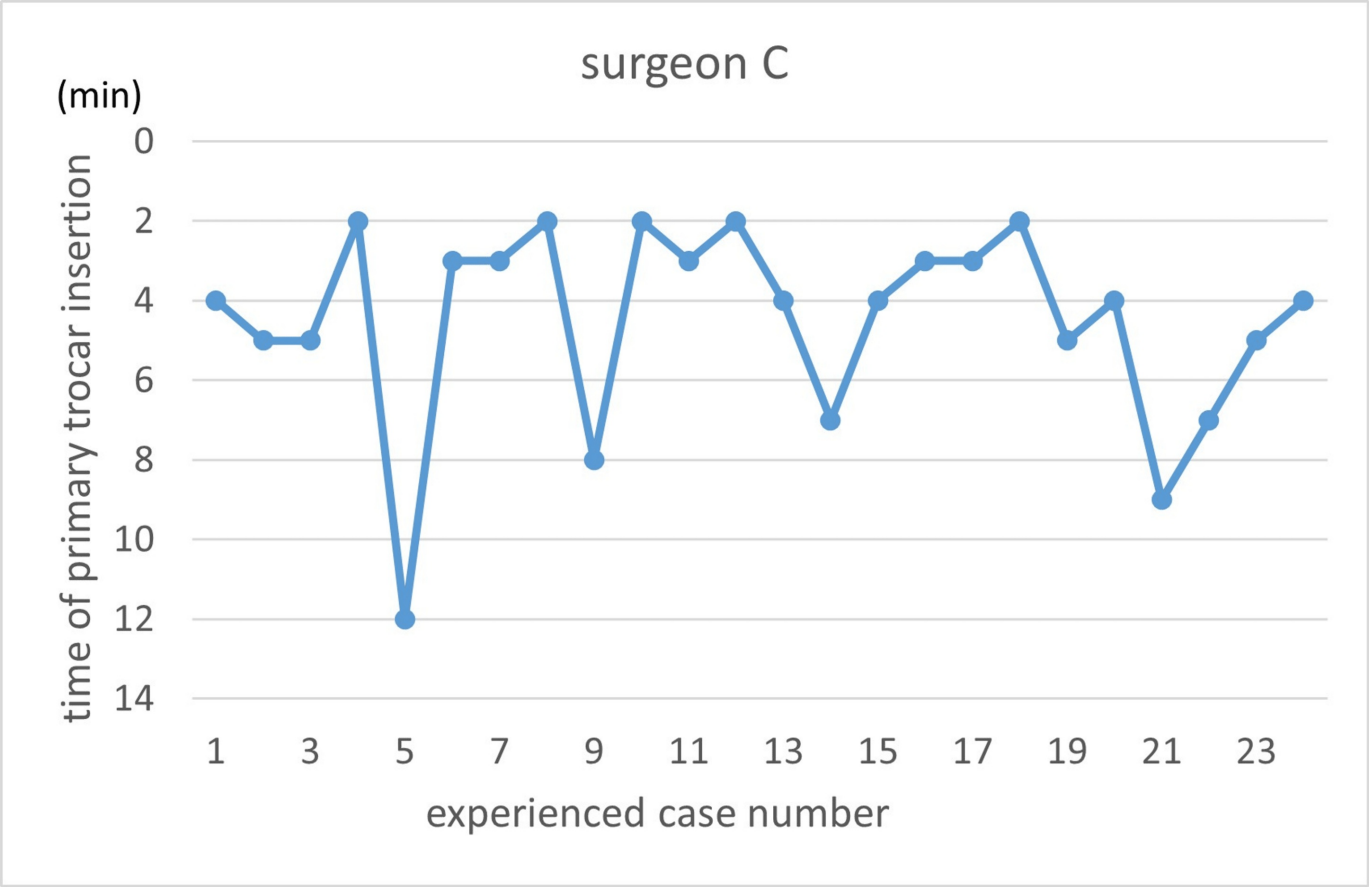
Learning curve of Surgeon C

**Figure 6:**
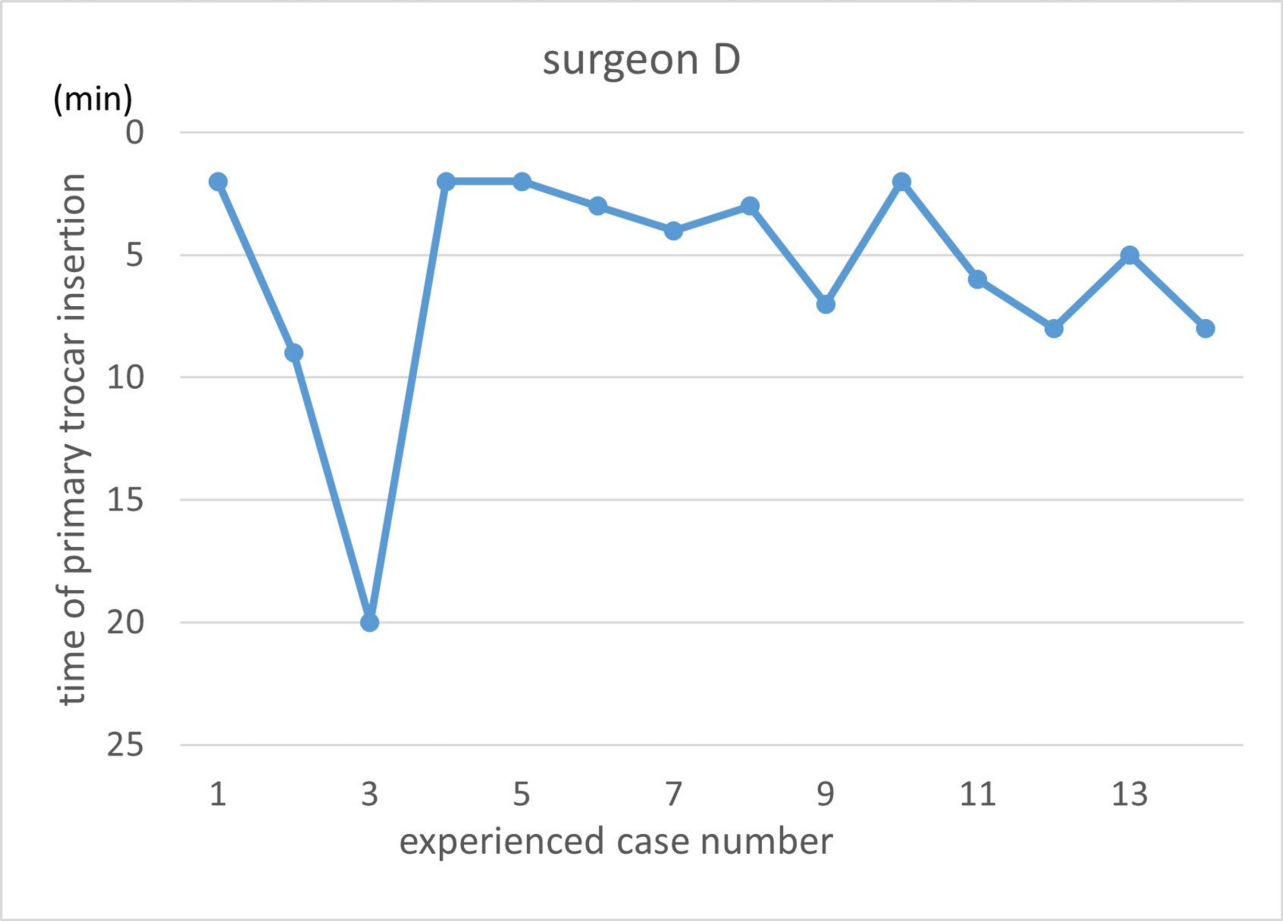
Learning curve of Surgeon D

**Figure 7:**
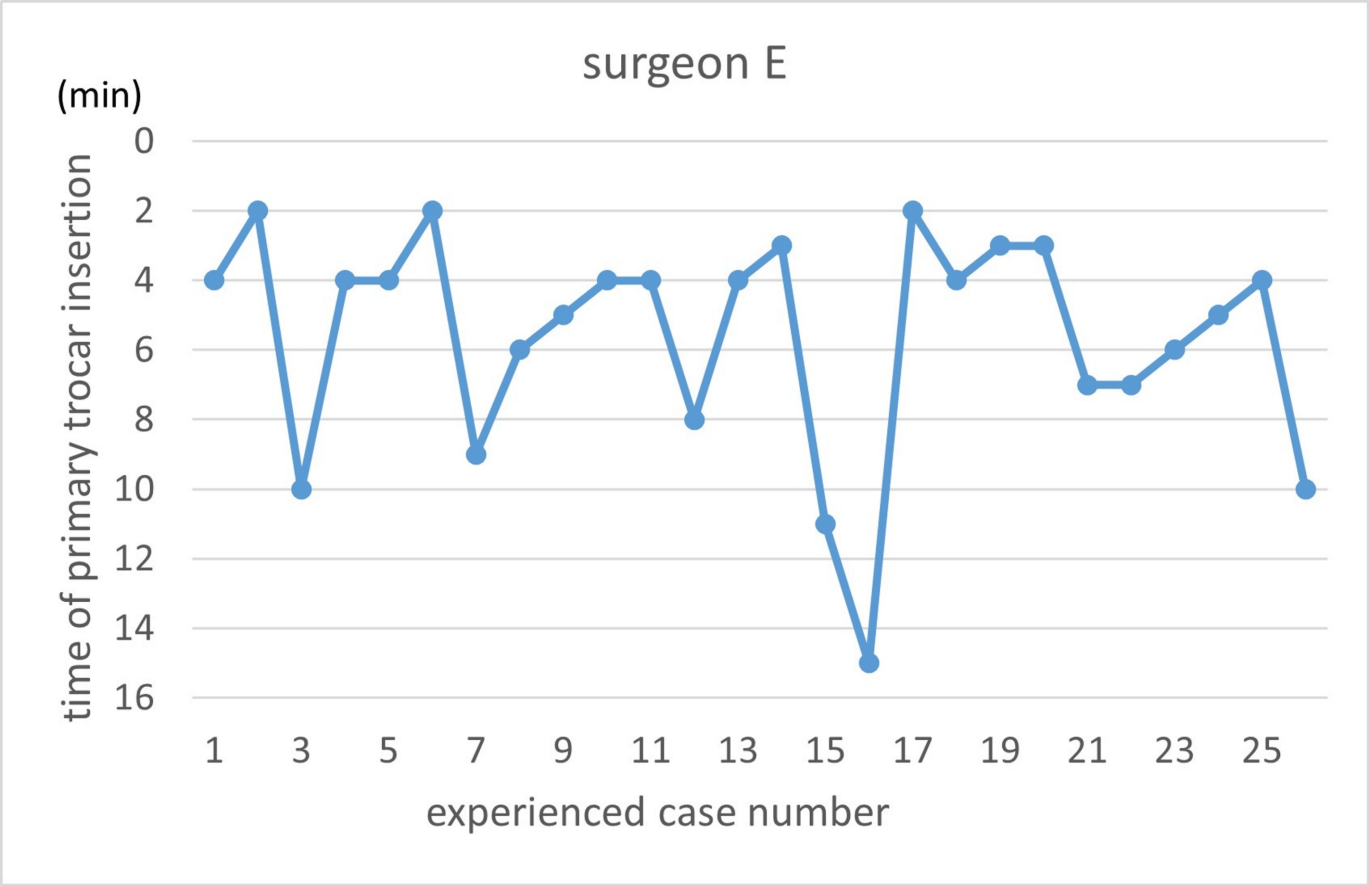
Learning curve of Surgeon E

Learning curves are illustrated using the primary trocar insertion time (Figures 3-7). Primary trocar insertion time is plotted on the y-axis, with a gradual decrease reflecting procedural familiarization, while the x-axis represents the cumulative number of cases performed during the study period. Although learning curves were illustrated for each surgeon, no statistical comparison between surgeons was conducted.

Unadjusted, descriptive outcomes and anatomical parameters

Unadjusted descriptive comparisons between the normal-weight and obese groups are shown in Table 3. In the normal-weight group, the median time of primary trocar insertion was four minutes (range, 1-21), with a mean of 5.1 ± 3.3 minutes. In the obese group, the median time of primary trocar insertion was six minutes (range, 2-20), with a mean of 7.0 ± 4.6 minutes.

**Table 3:** Unadjusted descriptive outcomes and anatomical parameters Anatomical parameters (subcutaneous fat thickness and umbilical wall thickness) were compared using the Mann–Whitney U test. Procedural times (primary trocar insertion time and wound closure time) are presented descriptively only, without inferential statistical testing, due to potential surgeon-related confounding and variability in closure technique. SD: standard deviation

Variables		Normal-weight group (n=65)	Obese group (n=20)	U-value	p-value
Time of primary trocar insertion (minutes)	Median (range)	4 (1-21)	6 (2-20)	-	-
	Mean±SD	5.1±3.3	7.0±4.6		
Wound closure time (minutes)	Median (range)	15 (9-26)	17 (10-30)	-	-
	Mean±SD	15±4.0	18±3.9		
Thickness of subcutaneous fat tissue (mm)	Median (range)	20 (7-44)	35 (15-51)	120	<0.01
	Mean±SD	21±6.4	35±8.8		
Thickness of umbilical wall (mm)	Median (range)	10 (3-31)	21 (6-45)	202.5	<0.01
	Mean±SD	11±5.9	22±9.7		

At this stage, primary trocar insertion time was summarized descriptively without inferential statistical testing because surgeon-related factors were considered potential confounders; this outcome was subsequently analyzed using a mixed-effects model. In addition, wound closure time was not subjected to inferential statistical testing because the closure technique varied among surgeons and was therefore described descriptively only.

Regarding the thickness of subcutaneous fat tissue, the median (range) was 20 (7-44) mm in the normal-weight group and 35 (15-51) mm in the obese group. The thickness of subcutaneous fat tissue was significantly greater in the obese group (p < 0.01). In terms of umbilical wall thickness, the median (range) was 10 (3-31) mm in the normal-weight group and 21 (6-45) mm in the obese group. The thickness of the umbilical wall was significantly greater in the obese group (p < 0.01).

Mixed-effects analysis adjusting for surgeon and case order

Because the primary trocar insertion time may be influenced by the surgeon as a confounding factor, a mixed-effects model was used with primary trocar insertion time as the dependent variable. Given the limited sample size, BMI and cumulative surgical experience (case order) were included as fixed effects. Surgeon was included as a random effect (categorical variable) to account for potential confounding due to inter-surgeon differences. In the mixed-effects linear model adjusting for surgeon and case order, BMI showed a trend toward longer primary trocar insertion time (estimate, 0.17 minutes per unit increase; 95% CI, −0.01 to 0.34; p = 0.06), whereas case order was not significantly associated with primary trocar insertion time (estimate, −0.075 minutes per case; 95% CI, −0.17 to 0.02; p = 0.11) (Table 4). The 95% CIs were calculated using standard errors derived from the mixed-effects model.

**Table 4:** Mixed random effect model analysis

Variables	Estimate	Standard error	95%CI	p value
Intercept	2.56	2.1	-	-
BMI	0.17	0.087	-0.01～0.34	0.06
Case order	-0.075	0.047	-0.17～0.02	0.11

Correlation between anatomical factors and trocar insertion time

**Figure 8:**
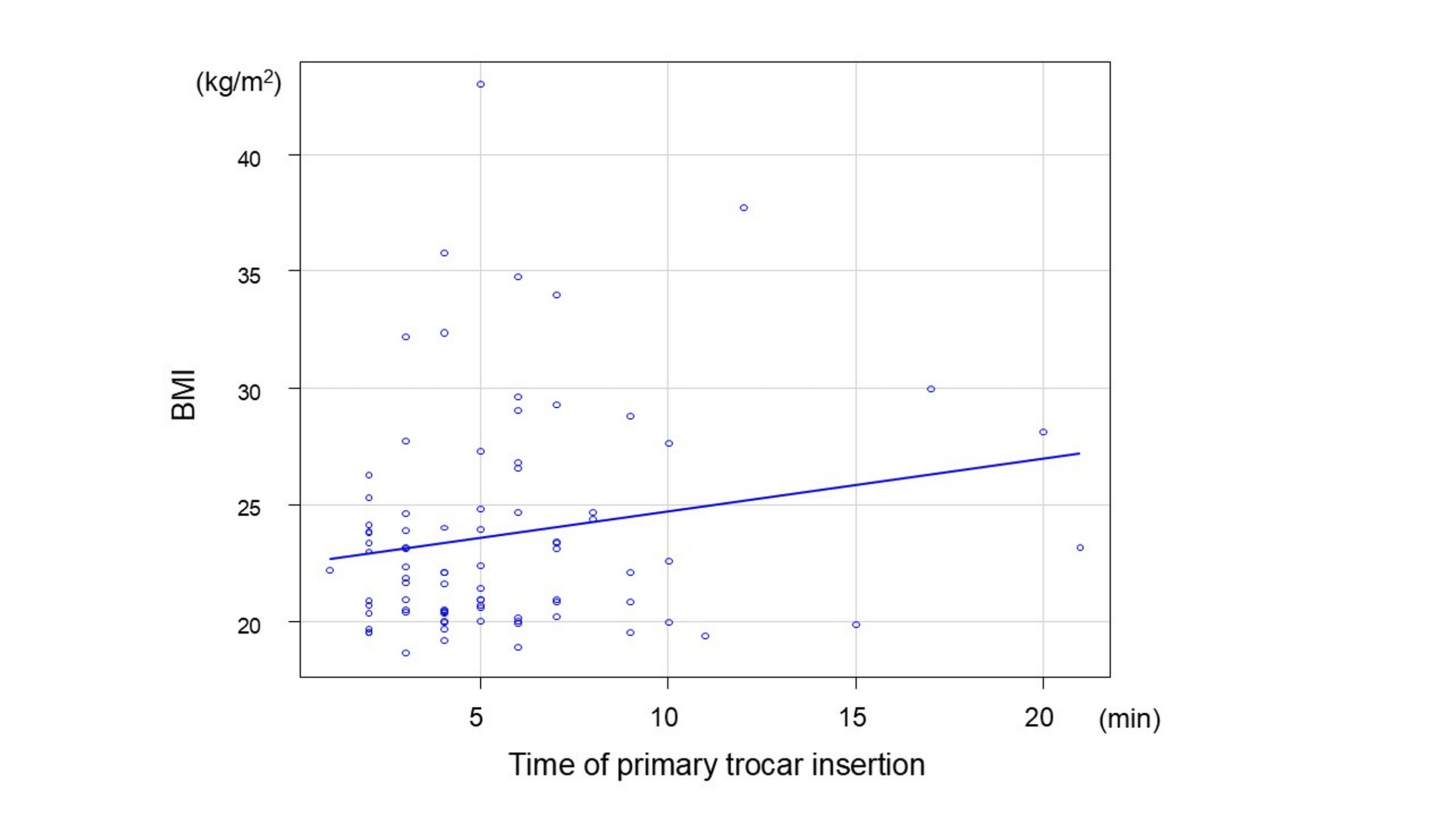
Correlation of BMI and time of primary trocar insertion The Spearman’s rank correlation coefficient was 0.14 (p = 0.203) BMI: body mass index

As an exploratory analysis, correlations among port insertion time, wound closure time, subcutaneous fat thickness, and umbilical wall thickness were assessed using Spearman’s rank correlation coefficient. Spearman’s correlation analysis was performed for all of 85 patients. Figure 8 shows the correlation between BMI and the time of primary trocar insertion. The Spearman’s rank correlation coefficient was 0.14 (p = 0.203), indicating no significant correlation.

**Figure 9:**
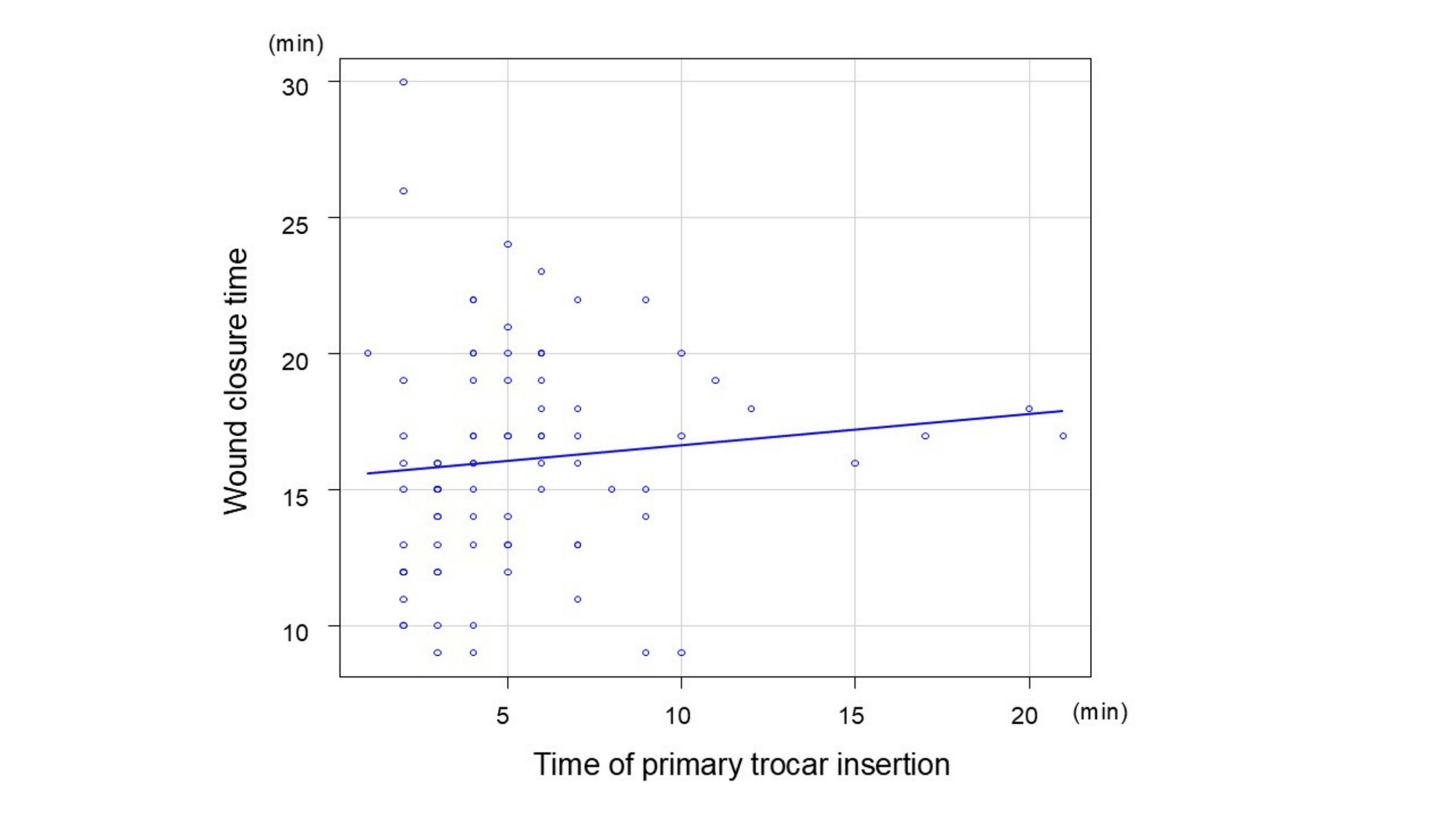
Correlation of time of primary trocar insertion and wound closure time The Spearman’s rank correlation coefficient was 0.24 (p <0.05)

Figure 9 shows the correlation between the time of primary trocar insertion and wound closure time. The Spearman’s rank correlation coefficient was 0.24 (p <0.05), indicating a weakly significant correlation.

**Figure 10:**
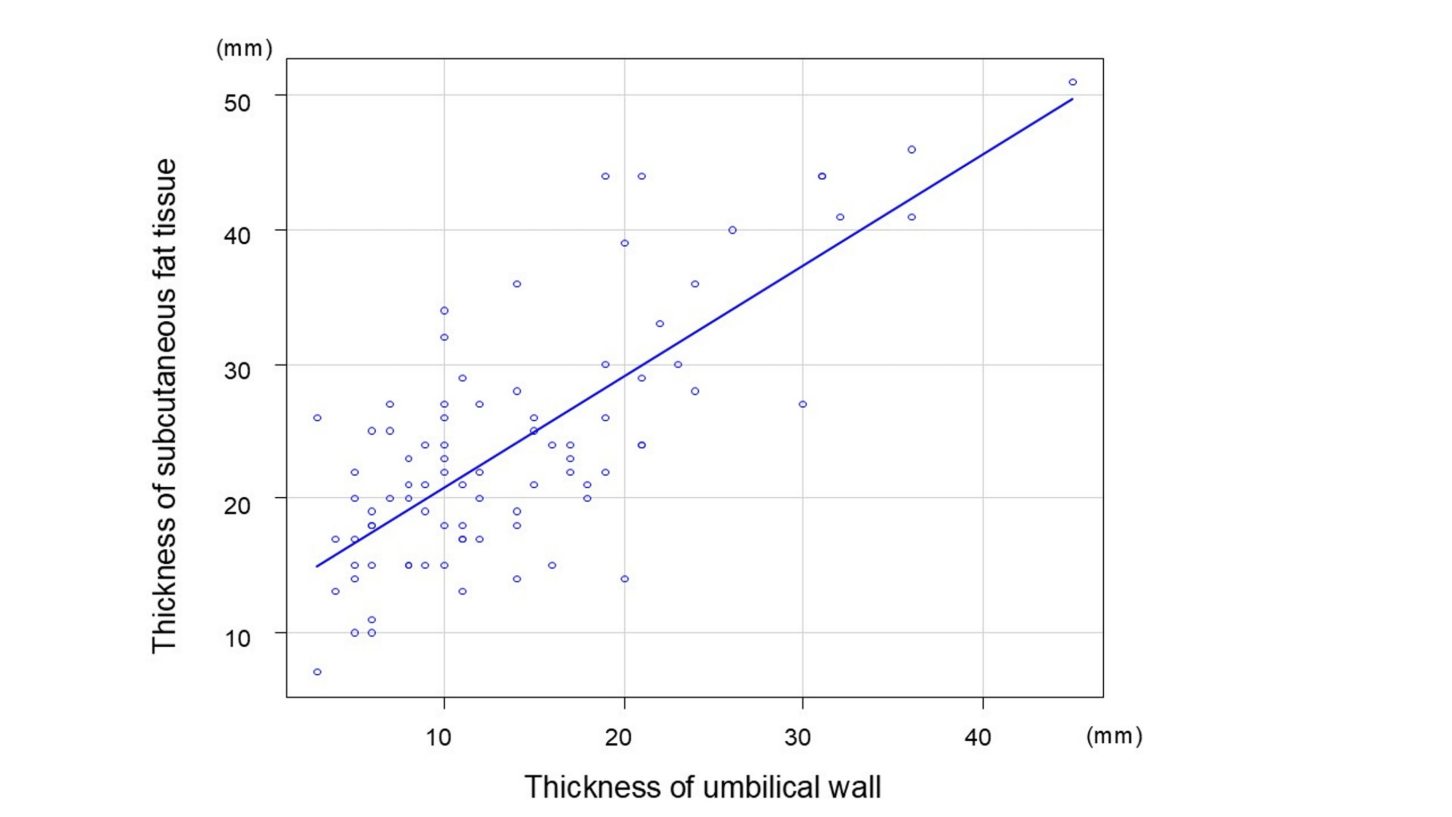
Correlation of thickness of subcutaneous fat tissue and umbilical wall The Spearman’s rank correlation coefficient was 0.63 (p<0.01)

Figure 10 shows the correlation between the thickness of subcutaneous fat tissue and the thickness of the umbilical wall. The Spearman’s rank correlation coefficient was 0.63 (p<0.01), indicating a moderately significant correlation.

**Figure 11:**
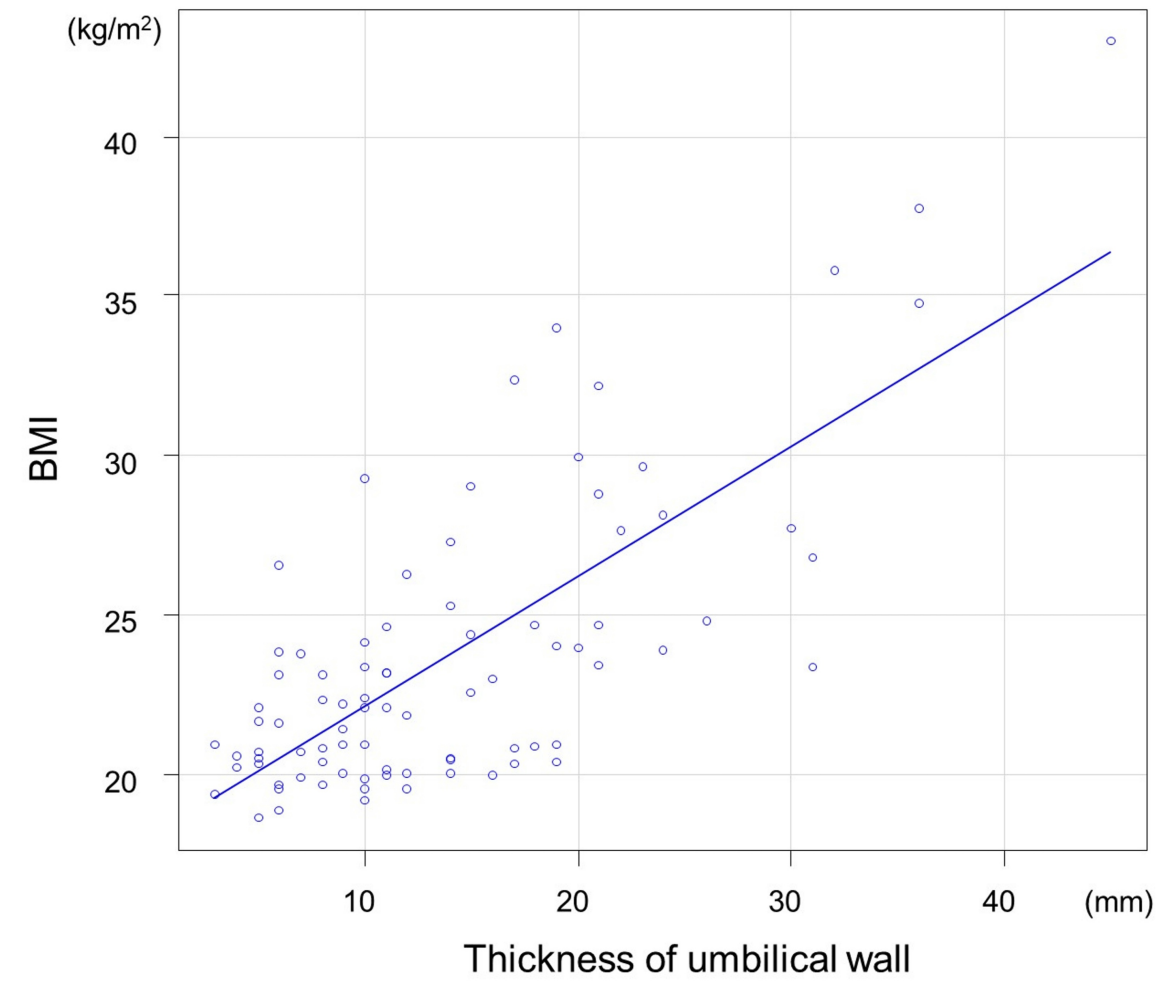
Correlation of BMI and thickness umbilical wall The Spearman’s rank correlation coefficient was 0.6 (p<0.01)

Figure 11 shows the correlation between BMI and the thickness of the umbilical wall. The Spearman’s rank correlation coefficient was 0.6 (p<0.01), indicating a moderately significant correlation.

**Figure 12:**
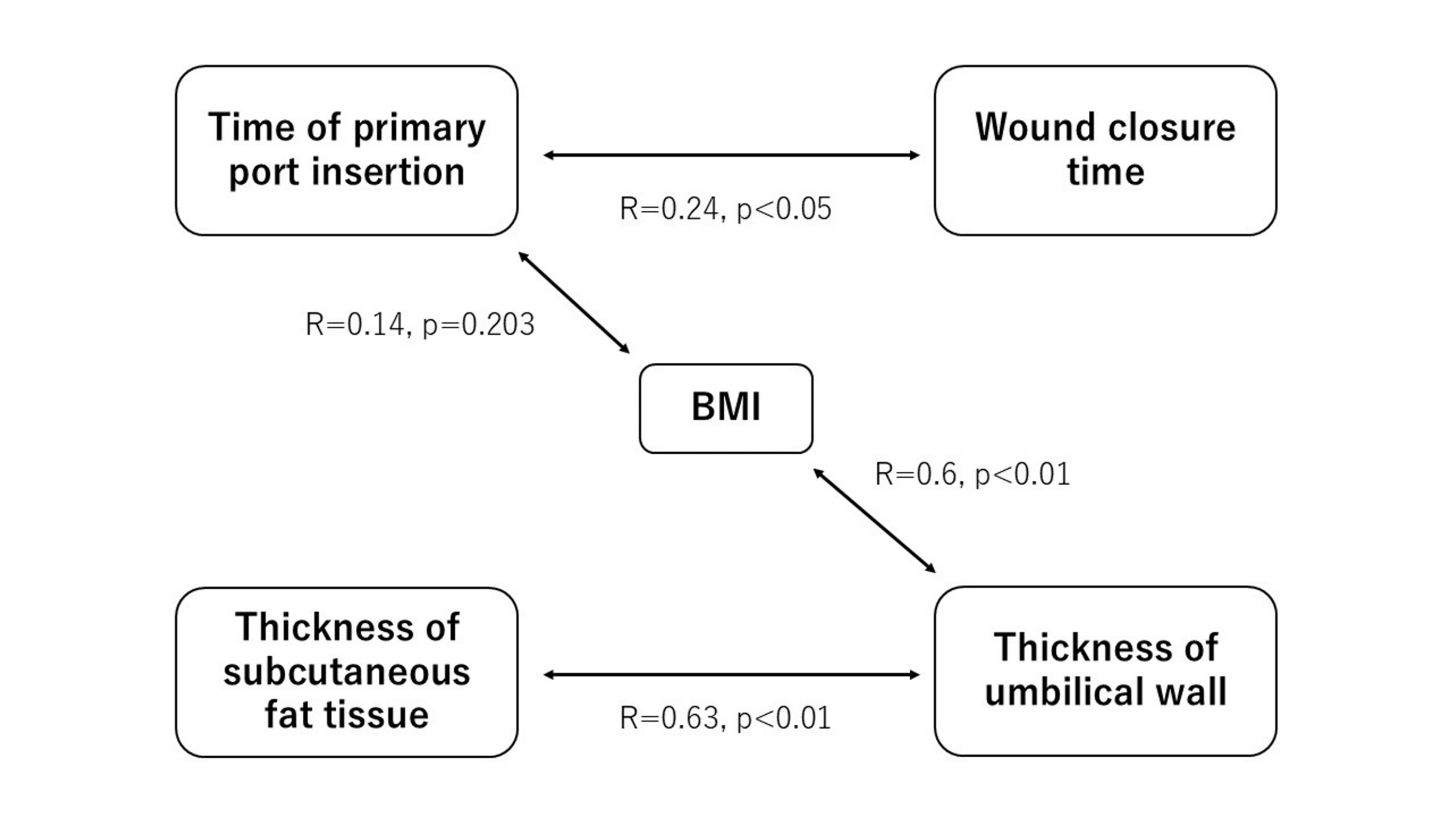
Summary of correlation

The associations among these parameters are summarized in Figure 12.

Outcome of surgical complications

Postoperative complications are summarized in Table 5.

**Table 5:** Postoperative complications of the two groups The outcome variables were categorical variables and were analyzed using Fisher’s exact test. NA: not available

Complications	Normal-weight group (n=65), n (%)	Obese group（n=20), n (%)	p-value
Complication	7 (10.8%)	4 (20%)	0.28
Bleeding over 500 ml	1 (1.5%)	0 (0%)	>0.99
Postoperative rebleeding	1 (1.5%)	0 (0%)	>0.99
SSI	2 (3%)	3 (15%)	0.082
Conversion to laparotomy	1 (1.5%)	0 (0%)	>0.99
Injury of omentum	0 (0%)	1 (5%)	0.24
Urinary tract injury	0 (0%)	0 (0%)	NA
Temporary lower limb impairment	1 (1.5%)	0 (0%)	>0.99
Ileus	1 (1.5%)	0 (0%)	>0.99
Acute kidney injury	1 (1.5%)	0 (0%)	>0.99

Complications occurred in seven patients (10.8%) in the normal-weight group (n=65) and in four patients (20%) in the obese group (n=20), with no statistically significant difference between the groups (p=0.28).

In the normal-weight group, one case each of conversion to laparotomy and ileus, bleeding during surgery over 500 mL, postoperative rebleeding, temporary lower limb impairment, and acute kidney injury (AKI) were observed, along with two cases of SSI of the umbilicus. In the obese group, complications included three cases of SSI of the umbilicus and one case of omental injury associated with port insertion. Urinary tract injury did not occur in either group.

The case of conversion to laparotomy was a 45-year-old female in the normal-weight group. The indication for surgery was pelvic pain and pelvic abscess due to endometriosis. The patient had undergone laparoscopic surgery for an endometriotic cyst 14 years earlier. The time of primary port insertion in this case was seven minutes. Although a complication did not occur during port insertion, because severe adhesions were found between the sigmoid colon and a left ovarian endometriotic cyst, an iatrogenic sigmoid colon injury occurred during adhesiolysis. The general surgery team performed a primary repair of the sigmoid colon, and stoma creation was avoided. The postoperative course was complicated by ileus, requiring bowel rest. The patient was discharged on postoperative day seven. This patient experienced two complications: conversion to laparotomy and ileus, which were counted separately in Table 5.

The patient who developed AKI was a 65-year-old woman in the normal-weight group who underwent bilateral salpingo-oophorectomy for an ovarian serous cyst. AKI was induced due to frequent use of NSAIDs while in a postoperative dehydrated state. After restricting the use of NSAIDs and administering diuretics, her renal function recovered.

The patient who developed pneumonia was a 56-year-old woman in the normal-weight group who underwent bilateral salpingo-oophorectomy for an ovarian serous cyst. She had a low-grade fever of 37°C and a cough preoperatively. In the postoperative course, she developed a fever of 38°C with a persistent cough. A CT scan revealed pneumonia in the right middle and lower lung fields. Her symptoms improved with antibiotic therapy, and she was discharged on postoperative day four. Haemophilus influenzae was later identified from a sputum culture. Although mild pneumonia may have been present preoperatively, it is possible that the surgical intervention triggered the clinical manifestation of the condition. 

All SSI cases were infections of the umbilicus and resolved with conservative management. The patient with lower-extremity neuropathy also recovered spontaneously. The patient with omental injury had a BMI of 37 and no history of prior abdominal surgery. She had a left ovarian endometriotic cyst, and the umbilical thickness was 36 mm. Intraoperatively, the bleeding site was controlled with bipolar coagulation.

## Discussion

In this retrospective cohort study, we examined the association between obesity and laparoscopic access-related outcomes using the open (Hasson) technique. Patients with obesity demonstrated significantly greater subcutaneous fat tissue thickness and umbilical wall thickness, confirming expected anatomical differences associated with higher BMI. Within the observed cohort, no statistically significant differences were detected by a mixed-effects model analysis in terms of BMI. However, these findings should be interpreted cautiously and should not be construed as evidence that obesity has no effect on operative difficulty or safety.

The correlation between subcutaneous fat tissue thickness and umbilical wall thickness provides insight into the anatomical composition of the abdominal wall. However, conclusions regarding BMI-independent procedural efficiency are based primarily on operative time-related outcomes and perioperative results, rather than on the correlation analysis alone.

Reports focusing on primary trocar insertion time remain limited. A previous study involving 430 patients with a mean BMI of 23.4 ± 3.5 found no significant correlation between BMI and insertion time, while identifying a positive correlation between first trocar insertion time and abdominal fat tissue thickness. In that study, the open technique was used, and the reported mean insertion time was 48.7 seconds [8]. Although this study is one of the few that have addressed port insertion time, the potential confounding effect of surgeon-related factors was not analyzed or discussed, and differences in reported port insertion times across studies likely reflect variation in operational definitions and study objectives, rather than true differences in procedural difficulty.

In contrast, another report described a mean insertion time of approximately five minutes using the open method and found no significant difference between normal-weight and obese patients, although group-specific mean values were not provided [4]. The findings of the present study are broadly consistent with the latter report, but direct comparison is limited by differences in outcome definitions, measurement methods, patient populations, and surgical settings.

Time-based outcomes such as primary trocar insertion time are inherently influenced by surgeon-related factors, including technical skill, baseline proficiency, and team dynamics. In this study, mixed-effects models were applied to partially account for inter-surgeon variability and learning-curve effects by incorporating surgeon-level random effects and case order as covariates. After adjustment, neither BMI nor case order showed a statistically significant association with time-related operative outcomes. Nevertheless, statistical modeling cannot fully eliminate confounding related to individual surgeon skill or unmeasured aspects of operative performance, and surgeon experience therefore remains a major limitation that restricts causal interpretation.

With respect to the learning curve, it is generally expected that increasing procedural experience is associated with improved technical proficiency and reduced operative time. In the present study, however, no clear monotonic reduction in primary trocar insertion time with increasing case order was observed. This may reflect the limited number of cases per surgeon, heterogeneity in baseline experience, and the relatively short duration of the port insertion step itself, which may make learning-curve effects difficult to detect using time alone as an outcome.

Regarding the primary outcome measurement, the open technique followed a standardized institutional protocol across surgeons, and time measurements were performed by operating room staff using standardized clock management rather than by the operating surgeon, in order to minimize measurement bias. Nonetheless, primary trocar insertion time remains a surrogate parameter that may not fully capture procedural complexity or technical difficulty, and it does not directly translate into patient-centered clinical outcomes.

Although no statistically significant differences in complication rates were observed between the two groups, this finding must also be interpreted with caution. Prior large-scale studies have reported increased risks of surgical site infection and venous thromboembolism in patients with severe obesity, particularly at BMI thresholds ≥35-40 [9]. In the present study, patients with very high BMI were underrepresented, and the study was underpowered to detect differences in rare access-related complications. Accordingly, the absence of statistical significance should not be interpreted as evidence of equivalent safety.

Taken together, this study should be regarded as exploratory and hypothesis-generating. While laparoscopic access using the open technique may be achievable in selected patients with obesity under standardized procedures, the primary contribution of this study lies in integrating objective anatomical measurements with operative parameters. These data may help inform the design of future prospective studies with standardized outcome definitions, rigorous control of surgeon expertise, and adequate power to evaluate access-related safety and efficiency in patients with elevated BMI.

Limitations

This study has several important limitations. First, it was a retrospective, single-center investigation with a relatively small sample size, particularly in the obese group, limiting statistical power and generalizability. Second, although mixed-effects models were used to partially adjust for surgeon-related variability, surgeon experience and technical skill could not be fully measured or controlled for and therefore represent a major source of residual confounding. Third, the time of primary trocar insertion, while measured using standardized operating room clock management, remains a time-based surrogate outcome that is vulnerable to variability and does not fully reflect procedural complexity or clinical impact. Fourth, abdominal wall measurements were derived from both CT and MRI, and oblique imaging planes around the umbilicus may have affected measurement reproducibility. Finally, the study was not adequately powered to detect clinically meaningful differences in rare access-related complications.

## Conclusions

In this retrospective single-center cohort study, laparoscopic access using the open (Hasson) technique was performed in both patients with normal weight as well as those with obesity without a statistically significant prolongation of primary trocar insertion time within the observed sample. Although patients with obesity demonstrated significantly greater subcutaneous fat tissue and umbilical wall thickness, these anatomical differences were not clearly associated with increased technical difficulty in primary trocar insertion after consideration of surgeon-related factors. However, these findings should not be interpreted as evidence of procedural equivalence or absence of clinically meaningful effects.

Given the observational design, limited sample size, and the potential for residual confounding, particularly related to operator experience, our results represent preliminary associations rather than causal conclusions. Nevertheless, within the constraints of this study, the data suggest that laparoscopic access via the open technique may be achievable in selected overweight and grade I obese patients when performed using a standardized technique and careful preoperative assessment. From a practical standpoint, these findings may help challenge the perception that elevated BMI alone should preclude minimally invasive access, while underscoring the importance of surgeon training and experience in access-related outcomes. Future prospective studies with adequate power, standardized and clinically meaningful outcome measures, and explicit control of surgeon expertise are required to validate these observations and to refine evidence-based recommendations for laparoscopic access in patients with elevated BMI.
